# Space Debris In-Orbit Detection with Commercial Automotive LiDAR Sensors

**DOI:** 10.3390/s24227293

**Published:** 2024-11-14

**Authors:** Isabel Lopez-Calle

**Affiliations:** Department of Electronics and Systems Engineering, University of Cadiz, 11519 Puerto Real, Spain; isabel.lopez@uca.com

**Keywords:** space debris, LiDAR, mapping, LASER, sensors

## Abstract

This article presents an alternative approach to detecting and mapping space debris in low Earth orbit by utilizing commercially available automotive LiDAR sensors mounted on CubeSats. The main objective is to leverage the compact size, low weight, and minimal power consumption of these sensors to create a “Large Cosmic LiDAR” (LCL) system. This LCL system would operate similarly to a giant radar circling the Earth, with strategically positioned LiDAR sensors along the target orbit. The article examines the feasibility of this concept by analyzing the relative orbital velocity between the sensor and debris objects, and calculating the time required to scan a complete orbit.

## 1. Introduction

Space debris refers to artificial objects in Earth’s orbit that no longer serve an operational purpose. These objects can vary in size, from small particles to defunct satellites or abandoned rockets. The issue of space debris has been growing over the decades as a result of the increase in space activities. The increase in space debris is primarily due to the proliferation of satellites in orbit, commercial and military space launches, and accidental collisions in space. The remnants of rockets and deorbited satellites also contribute to debris [1]. Space debris poses a significant risk to space missions and the International Space Station. Collisions with space debris can damage or destroy operational satellites and generate more fragments [2,3,4]. The accumulation of space debris also raises environmental concerns. Various strategies have been proposed to manage and mitigate space debris, including active cleanup through technologies such as nets or robotic arms, and improving guidelines for the safe disposal of satellites at the end of their operational life [5,6].

The scientific and space community continually conducts research on space debris, including data collection and tracking of objects in orbit, with the aim of better understanding the problem and developing effective solutions [7,8,9]. At present, ground-based RADAR and LiDAR are the main research tools that contribute to space debris cataloging databases [10,11,12,13]. However, only 10% of this debris has been detected [12], as most of it consists of very small particles, below 10 cm in diameter, and becomes less detectable under the influence of the atmosphere.

In contrast, space-based optical imaging is another approach for detecting and tracking space debris, as it avoids environmental constraints such as atmospheric interference. This method enables capturing high-resolution images and uses advanced imaging algorithms to effectively enhance the resolution and detectability of space debris [14,15,16]. However, these techniques come with significant challenges, including the complexity of deploying high-resolution cameras in orbit, the increase in payload weight, and the intensive data processing required, which leads to a high power consumption.

In this context, emerging and innovative technologies are being explored for the detection and tracking of space debris. In conclusion, this article is based on the novel concept of using commercial automotive LiDAR sensors, which were originally developed by the automotive industry to enable autonomous vehicles to detect and avoid objects. Because of their compact size, low weight, and minimal power requirements, these sensors could be deployed onboard CubeSats to detect space debris in Earth’s orbit.

Although LiDAR sensors are widely used in various terrestrial applications and are also being used in space for Earth-observation purposes, their potential for space debris in-orbit detection remains largely unexplored. The aim of this work is to propose a novel application of LiDAR technology to measure the density of space debris that pose significant risks to current and future space operations [17]. Although this study does not discuss the technical specifications of the LiDAR sensors themselves, it focuses on how such sensors can be integrated into small satellites to enhance debris tracking capabilities. The use of LiDAR in this specific context has not been widely discussed or implemented to date, and its application could provide critical insights for the management of space debris.

LiDAR (Light Detection and Ranging) is similar to microwave RADAR (Radio Detecting and Ranging) in remote sensing, but with much shorter wavelengths (optical or infrared). LiDAR is an active sensor (laser or led) that emits an electromagnetic pulse and receives the signal that bounces back with a photo/iR detector. Because of the much shorter wavelength, lasers can be modulated much faster and the laser beams can be collimated to a much smaller spot. As a result, LiDARs generally have much higher temporal and spatial resolutions and smaller instrument sizes [18]. Unlike ground-based tools, LiDAR systems on satellites are not hindered by atmospheric conditions [19], offering a more effective and direct means of monitoring space debris.

LiDAR was first used primarily in aerospace and topographic applications because of its high cost and size. With the rise of autonomous vehicle technology, LiDAR has gained importance in providing vehicles with a 3D perception of the environment [20]. Efforts have been made to miniaturize LiDAR components to make them more streamlined and aesthetically acceptable in vehicles. The evolution of LiDAR systems in the automotive field is continuously progressing, with significant advances in miniaturization, costs, and technical capabilities.

## 2. Materials and Methods

**Figure 1:**
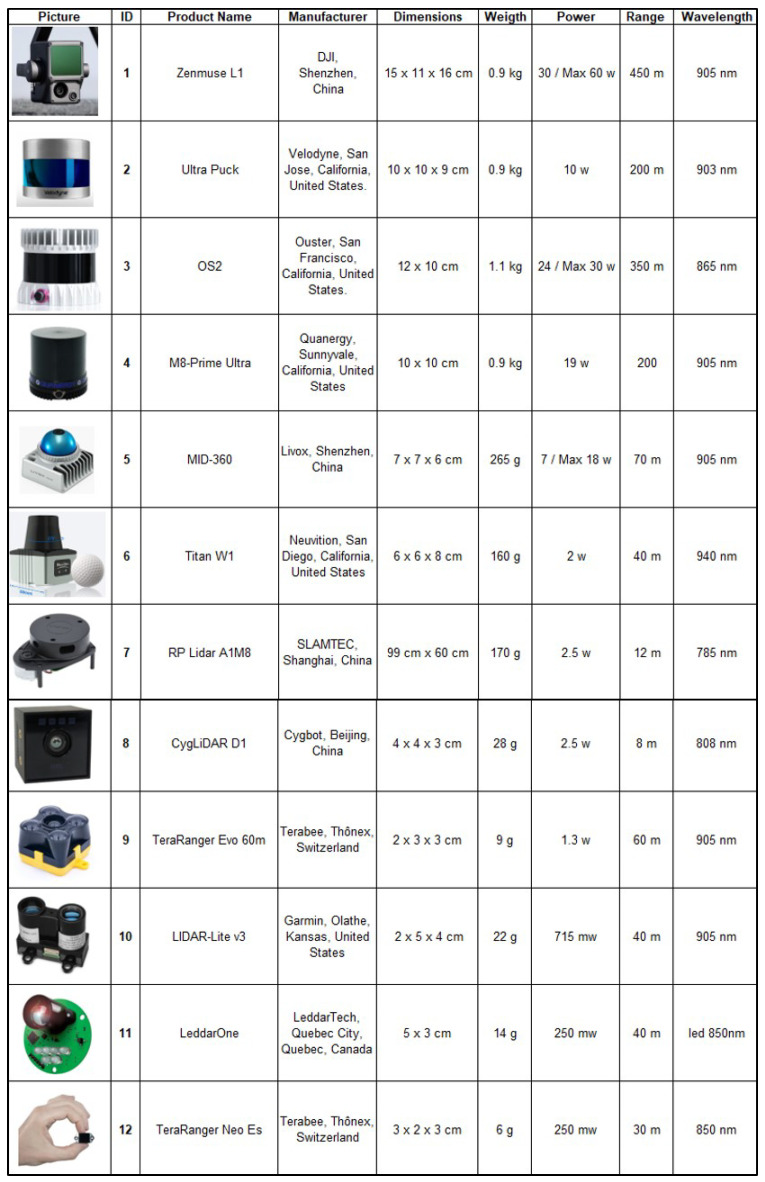
Commercial Automotive LiDAR Sensors.

Figure 1 presents the key characteristics of the LiDAR sensors currently available on the market that are suitable for installation on small satellites, including their lightweight, compact size, and low power consumption. For this reason, only short-range sensors are included in this table. Short-range proximity LiDARs can support up to 500 m with one- (1D) or three-dimensional (3D) imaging. Long-range LiDAR configurations can support up to 1000 km range, but their power consumption, size, and weight are too substantial for integration onto a small satellite or CubeSat.

Thus, if we use an automotive LiDAR sensor onboard a satellite, on the one hand, we eliminate the presence of clouds, thus avoiding their major disadvantage for particle detection from the ground. On the other hand, we take advantage of its light weight, dimensions, and power consumption to use it in particle detection directly in the vicinity of the satellite.

### Maximum Distance from LiDAR to Object at Orbital Velocities

The initial focus is on determining the effective range distance from which we can reliably detect an object with the orbital velocity of the sensor.

As all objects that orbit the Earth must maintain a specific relationship between altitude and orbital velocity, it is possible to calculate the relative velocity between two objects that share the same orbital parameters and are separated by a radial distance *R*, which represents the sensor’s detection range. This maximum distance between objects is characterized by Equation (1):

(1) $$\begin{array}{c} r_{object}-r_{lidar}=R \end{array}$$

where $r_{object}$ is the distance from the object to the center of the Earth and $r_{lidar}$ is the distance from the LiDAR sensor onboard the satellite to the center of the Earth.

**Figure 2:**
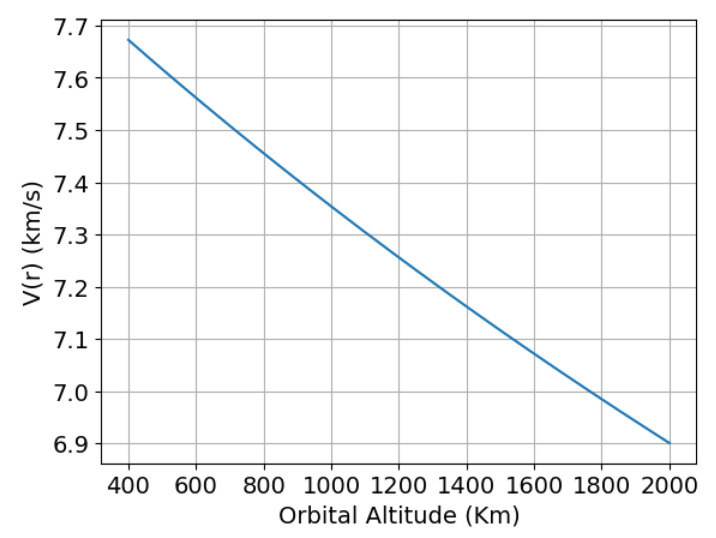
Orbital velocity *V(r)* as a function of the distance r to the Earth’s surface (LEO orbits).

Equation (2) shows the relationship between the orbital velocity *V(r)* and the distance r from the centre of Earth, where *G* is the Newton’s gravitational constant and *M* is the Earth’s mass. Figure 2 illustrates how the orbital velocity *V(r)* varies inversely with distance from the Earth’s centre, according to the principle that the orbital velocity decreases as an object moves far away from Earth’s gravitational influence.

(2) $$\begin{array}{c} V\left(r\right)=\sqrt{\frac{GM}{r}} \end{array}$$

For example, with an orbit altitude of *r* = 840 km, the sensor velocity is around 7437.8654 m/s, and the relative velocity between the object and the sensor, located at *R* = 50 m far away from the sensor (*R* is the sensor range), is 0.26 m/s or around 1 km/h (expressed with the common units of speed of a car).

In order to be able to detect the object, the velocity of the detection system, defined by the signal integration speed, must be equal or greater than the relative velocity between the sensor and the object, so that the signal integration speed of all commercial LiDARs is higher than the relative low velocity between the object and the sensor with a range distance between them, enabling the commercial automotive sensor to detect the object even at orbital velocities.

We consider the LiDAR sensor rotating around the Earth in a circular orbit, and the object rotating with the same orbit parameters as the sensor, but at an altitude equal to the sensor’s altitude plus the sensor’s range of view. The simplest form of a LiDAR sensor (1D) can only detect objects directly perpendicular to its motion within its field of view. Consequently, the object becomes visible to the sensor only when it passes directly in front of it. With a relative velocity of 1 km/h between the LiDAR and the object (assuming that the LiDAR has a range of vision of 50 m), this means that the sensor is moving 1 km/h faster than the object. Therefore, if the object is located 1 km away, it will take one hour for the sensor to detect it.

Following the same reasoning, imagine that two objects orbit the Earth at different altitudes characterized by the angular velocity.

- Object 1—LiDAR: orbiting at an altitude of 840 km.
- Object 2—debris: orbiting slightly higher, at 840.05 km.

The relative angular velocity between the sensor and object is crucial to understand the total orbit scanning time, in order to detect all the objects placed on the target orbit.

The objects move at slightly different angular velocities because of their altitude difference. The relative angular velocity is defined in Equation (3):

(3) $$\begin{array}{c} \Delta \varpi =(\varpi_{lidar}-\varpi_{object}) \end{array}$$

To calculate the time required for object 1 (the LiDAR sensor) to catch up to object 2, placed at the maximum possible angular distance from the sensor, which is 360 degrees, we calculate the time needed for object 1 to cover this distance at the relative angular speed.

Thus, the total orbit scanning time is defined in Equation (4).

(4) $$\begin{array}{c} \mathrm{Scanning}\;\mathrm{Time}\;\mathrm{per}\;\mathrm{Orbit}=\frac{2\pi }{\Delta \varpi } \end{array}$$

In the example, with object 2 being just 50 m higher, the sensor for object 1 would catch up to object 2 in approximately 18.6 years.

**Figure 3:**
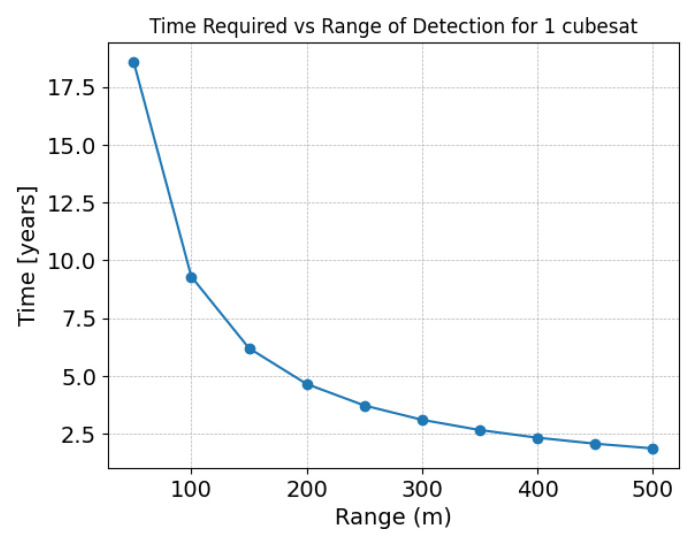
Time required to scan the complete orbit vs. the sensor’s detection range at 840 km altitude.

In Figure 3, there is a graphical representation of the time needed to scan the entire orbit as a function of the sensor’s range or distance to the object. In this figure, you can clearly see something that is paradoxical from an Earth-based perspective: the total scan time decreases as the object is farther away. In other words, it takes longer to see the objects that are closer than the objects that are farther away from the sensor.

At this point, we observe how the time required to scan a single orbit can become excessively long for the closest distances. Thus, the greater the sensor’s range, the less time is needed to scan a complete orbit located at the farthest distance from the sensor. However, if the sensor has a wider range, this means that the host satellite must be larger to meet the necessary requirements for power, size, and weight to ensure the operability of the sensor.

Therefore, another approach to reduce the scanning time of a complete orbit with a short range sensor is to deploy multiple sensors along the same orbit. By doing so, we effectively decrease the scanning time proportionally to the number of sensors scanning the orbit and distributed at an angular distance equivalent to the full orbit divided by the number of sensors.

Given the orbit example, where we are examining 840 km altitude with a sensor range of 50 m easily installable on a small sat or CubeSat, the time required to scan a complete orbit would be 1.85 years if we use 10 sensors in a constellation configuration.

**Figure 4:**
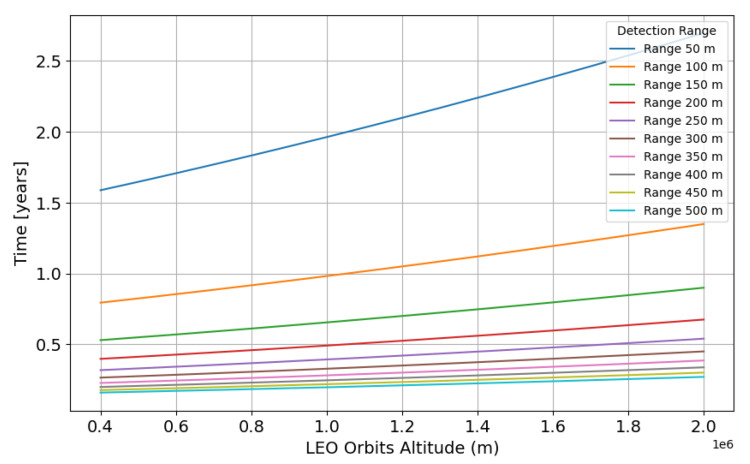
The number of years required for a complete scanning as a function of altitude with 10 LiDAR sensors per orbit.

In Figure 4, we show the scanning time for different low-Earth orbits (LEO) accessible to Cubesats, considering a constellation of 10 satellites for different detection ranges.

In Figure 4, it can be observed that for a typical LEO altitude of 1000 km, the orbital scanning duration is less than one year only for detection ranges above 100 m, which is is suitable for ID sensor’s 1 to 4, described in Figure 1.

## 3. Discussion

Deploying multiple satellites in the same orbit forming a constellation of satellites can reduce the overall scanning time.

**Figure 5:**
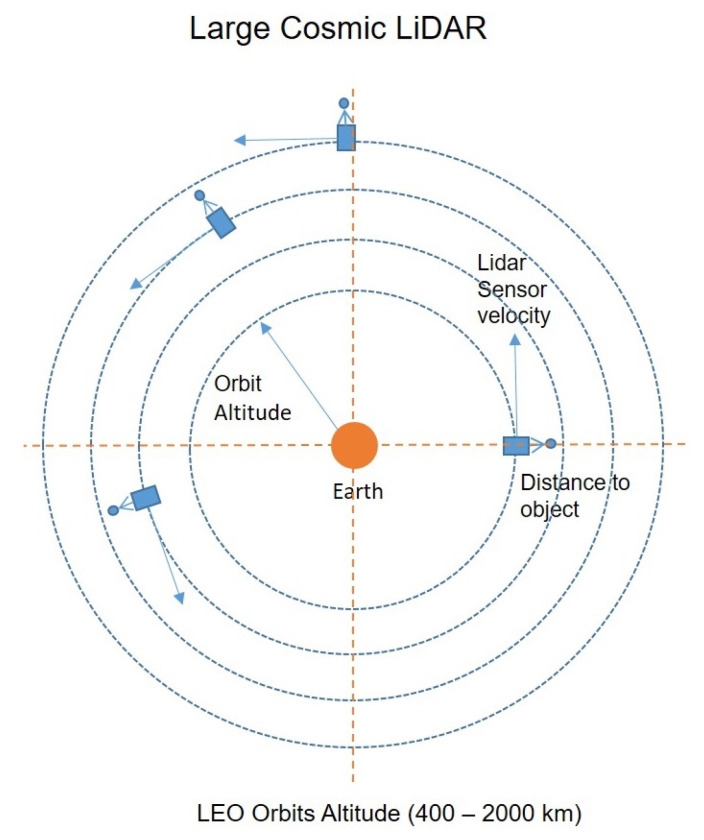
Conceptual design of a Large Cosmic LiDAR.

This full study introduces the new concept of a Large Cosmic LiDAR (LCL). There is a description of the conceptual design of a Large Cosmic LiDAR in Figure 5.

This concept can be compared to deploying an enormous radar system circling the Earth. However, instead of using traditional radar, the detection mechanism employs a LiDAR sensor—or constellation of LiDAR sensors—positioned at the sensor’s effective detection range within the target orbit. In a Large Cosmic LiDAR, the sensor onboard is able to scan the target orbit with the resolution of the LiDAR’s range.

The detection system outlined by LCL involves scanning the satellite’s orbit while maintaining a constant radial orientation. In this setup, the sensor faces outward from the orbit, with the satellite’s orientation sustained throughout by the ADCS (Attitude Determination and Control System) onboard the CubeSat.

Once all of the characterization of the detection system has been completed and according to the automotive LiDAR sensors currently in the market and summarized in Figure 1, a range below 500 m is quite common for LiDAR sensors with the requirements of being onboard of a small satellite: small size, weight, and power. These sensors can be identified in the table with IDs 1 to 12.

Based on the analysis conducted and the information known so far, the LCL system will be designed to achieve a mission duration of 0.5 years for each sensor, which is the typical duration for space missions in low orbits without propulsion systems.

Table 1 presents the LCL configuration for each sensor in Figure 1. It includes the Sensor ID, the range, and the number of satellites. The background color of each cell serves as a color code that classifies the LCL system according to the number of satellites in the constellation.

Table 2 explains the color code used in Table 1. The background color is green if the constellation has fewer than 5 satellites, orange if it falls between 5 and 10, purple if it is between 10 and 50, and red if it is more than 50.

The criterion for choosing the best configuration will depend on the launch possibilities and control of a large number of satellites equipped with the same sensor. The deployment of small satellites in orbit can be just as costly as establishing a single sensor if they can travel on the same launcher. The system becomes more complex if data must be collected from one or many satellites, but this has to be a decision between complexity and costs. There are many possible configurations. It is important to emphasize that detection methodology primarily targets orbital objects that share the same orbital inclination. For objects orbiting with inclinations that differ, traditional one-dimensional sensors are insufficient for detection. To effectively identify such objects, the implementation of three-dimensional sensors onboard or the addition of sensors on alternate CubeSat faces would be necessary.

If the satellite exhibits a spin frequency, the scanning also shifts from 1D to 3D. It should be noted that achieving a near 4pi steradian detection range would theoretically be possible, depending on the satellite’s rotational dynamics and sensor configuration. This would require the satellite to rotate so that the sensor field of view covers all directions, but would also significantly increase the complexity of the system due to the contribution of multiple spinning frequencies, the satellite spin, and the sensor spin.

The primary preference is to use a single sensor per CubeSat to streamline onboard data management and transmission, thereby reducing system complexity. This approach enables efficient handling of the sensor onboard a small satellite, ensuring minimal power consumption and simplified data management processes.

## 4. Conclusions

Our study aims to demonstrate the application of LiDAR sensors, similar to those used in autonomous vehicles, in the context of space applications. Following this conceptual demonstration, our intention is to validate the technology transfer of LiDAR sensors to address challenges in space debris detection, collision prevention, and debris management. Our mission serves the dual purpose of confirming the capabilities of these sensors and contributing to the information available in the space debris database published by space agencies. This effort is expected to foster growth in the space industry and in the tracking and surveillance sector.

The proposed LiDAR systems, deployed on a CubeSat or even in constellations of satellites, will bring a set of advantages in the study of the debris flux that make them stand out in the context of space debris detection and collision prevention. Their high precision in distance measurement, superior angular resolution, and all-weather operation make LiDAR-equipped CubeSats well-suited for continuous monitoring of LEO orbits. While LiDAR does not rely on ambient light like optical cameras do, it can operate effectively in various lighting conditions [21,22,23]. In addition, their flexibility, cost-effectiveness, and quick-response capabilities offer a dynamic and efficient approach to space debris management.

Advancements in technology, including the miniaturization of LiDAR components and their low power consumption, have made it feasible to integrate LiDAR on CubeSats, overcoming historical size and weight constraints. These advancements contribute to the adaptability of LiDAR technology for space debris monitoring.

## Figures and Tables

**Table 1:** Configuration of the LCL for each sensor in Figure 1 at an altitude of 840 km and scanning time of 6 months.

Sensor ID	1	2	3	4	5	6	7	8	9	10	11	12
Range (m)	450	200	350	200	70	40	12	8	60	40	40	30
Satellites	5	10	6	10	27	47	155	233	31	47	47	62

**Table 2:** Color code classification for the LCL system by number of satellites.

Color	Number of Satellites	Description
Green	<5	Low constellation
Orange	5–10	Moderate constellation
Purple	10–50	High constellation
Red	>50	Very high constellation

## Data Availability

Data are contained within the article.
